# Autophagy in Viral Infection and Pathogenesis

**DOI:** 10.3389/fcell.2021.766142

**Published:** 2021-10-15

**Authors:** Shan Liang, Yun-Shan Wu, Dong-Yi Li, Ji-Xin Tang, Hua-Feng Liu

**Affiliations:** ^1^Key Laboratory of Prevention and Management of Chronic Kidney Disease of Zhanjiang, Institute of Nephrology, Affiliated Hospital of Guangdong Medical University, Zhanjiang, China; ^2^Shunde Women and Children’s Hospital, Guangdong Medical University (Foshan Shunde Maternal and Child Healthcare Hospital), Foshan, China

**Keywords:** autophagy, xenophagy, virophagy, viral infection, innate immune response, antigen presentation, inflammation and immunity

## Abstract

As an evolutionarily conserved cellular process, autophagy plays an essential role in the cellular metabolism of eukaryotes as well as in viral infection and pathogenesis. Under physiological conditions, autophagy is able to meet cellular energy needs and maintain cellular homeostasis through degrading long-lived cellular proteins and recycling damaged organelles. Upon viral infection, host autophagy could degrade invading viruses and initial innate immune response and facilitate viral antigen presentation, all of which contribute to preventing viral infection and pathogenesis. However, viruses have evolved a variety of strategies during a long evolutionary process, by which they can hijack and subvert host autophagy for their own benefits. In this review, we highlight the function of host autophagy in the key regulatory steps during viral infections and pathogenesis and discuss how the viruses hijack the host autophagy for their life cycle and pathogenesis. Further understanding the function of host autophagy in viral infection and pathogenesis contributes to the development of more specific therapeutic strategies to fight various infectious diseases, such as the coronavirus disease 2019 epidemic.

## Introduction

**FIGURE 1:**
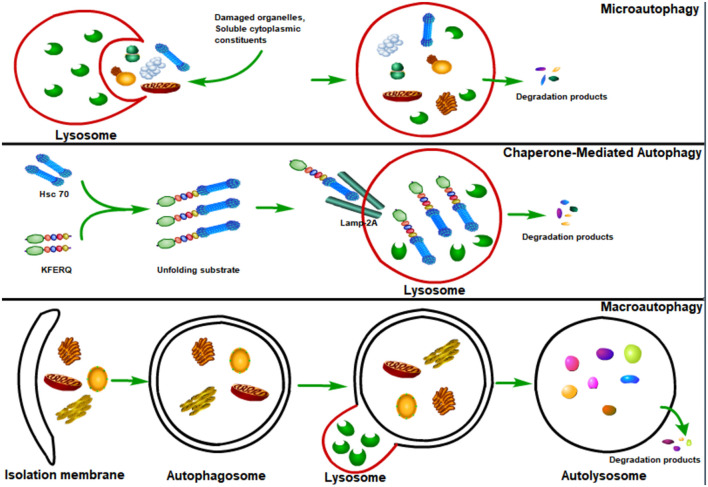
Major types of autophagy. According to the way that eukaryotic cells deliver cytoplasmic cargo to lysosomes for degradation, autophagy can be divided into three major types: macroautophagy, microautophagy, and chaperone-mediated autophagy (CMA). Microautophagy refers to the lysosome itself engulfing cytoplasmic material or large structures by invading the lysosome membrane. The CMA only degrades soluble proteins in a selective manner through the LAMP2A receptor on the lysosome to recognize and translocate unfolding proteins with a specific signal sequence—KFERQ. Macroautophagy could both selectively and non-selectively engulf bulk cytoplasmic components by sequestering these cargoes to a specialized double-membrane vesicle known as the autophagosome; autophagosome is then fused with the lysosome, where the cargo is degraded and the resulting macromolecules are released into the cytosol for reuse.

Autophagy, or cellular self-digestion, is an evolutionarily conserved cellular process through which long-lived proteins, damaged organelles, or invading pathogens could be degraded by the lysosome (Levine et al., 2011; Deretic et al., 2013; Levine and Kroemer, 2019; Mizushima and Levine, 2020). According to the way that eukaryotic cells deliver cytoplasmic materials to lysosomes for degradation, autophagy can be divided into three major types: microautophagy, chaperone-mediated autophagy (CMA), and macroautophagy (Mizushima et al., 2008; Figure 1). Microautophagy engulfs cytoplasmic materials or large structures through non-selectively invaginating lysosomal membrane or selectively delivering soluble cytosolic proteins to the multivesicular bodies (MVBs) (Mijaljica et al., 2011; Sahu et al., 2011). CMA only degrades soluble proteins in a selective manner through the lysosomal LAMP2A receptor to recognize and translocate unfolding proteins with a specific signal sequence—KFERQ (Orenstein and Cuervo, 2010; Kaushik and Cuervo, 2012). Macroautophagy could both selectively or non-selectively engulf bulk cytoplasmic components by sequestering these cargoes to a specialized double-membrane vesicle (DMV) known as the autophagosome (Feng et al., 2014; Melia et al., 2020; Nakatogawa, 2020). Here, we focus on macroautophagy, and hereafter refer to “macroautophagy” simply as “autophagy.”

Autophagy begins with the sequestration of cargoes into a cup-shaped double membrane known as the isolation membrane or phagophore, which stems from several cellular compartments (Shibutani and Yoshimori, 2014; Melia et al., 2020). The sequestration of cargoes could be either non-specific (such as the engulfment of bulk cytoplasm) or selective (such as specific engulfment of organelles or invading pathogens). The gradually expanding phagophore envelops the engulfed cargoes, forming the autophagosome, which then fuses with the lysosome, causing the formation of autolysosome (Hamasaki et al., 2013; Lamb et al., 2013). The lysosome provides hydrolases to the autolysosome, where the autophagosome inner membrane is lysed and the cargoes break down, and then the resulting macromolecules are released back into the cytosol for reuse through membrane permeases (Mizushima et al., 2008; Lamb et al., 2013).

Autophagy is involved in a variety of mammalian physiological processes such as the maintenance of energy homeostasis, cell differentiation and development, and innate immunity against invading pathogens (Kuma et al., 2004; Levine and Kroemer, 2008; Mizushima and Levine, 2010; Levine et al., 2011; Deretic et al., 2013). Given the powerful function to degrade intracellular substances, host autophagy is activated during the viral infection so as to degrade various invading viruses (Levine et al., 2011; Deretic et al., 2013). However, an increasing body of evidence suggests that viruses have developed various strategies to hijack and subvert the host autophagy for their life cycle and pathogenesis (Heaton and Randall, 2010; Levine et al., 2011; Deretic et al., 2013). In this review, we focus on the function of autophagy in the process of viral infection and pathogenesis and then discuss the mechanisms of how viruses usurp the host autophagy to facilitate their life cycle and pathogenesis.

## The Function of Autophagy in Antiviral Defense

**FIGURE 2:**
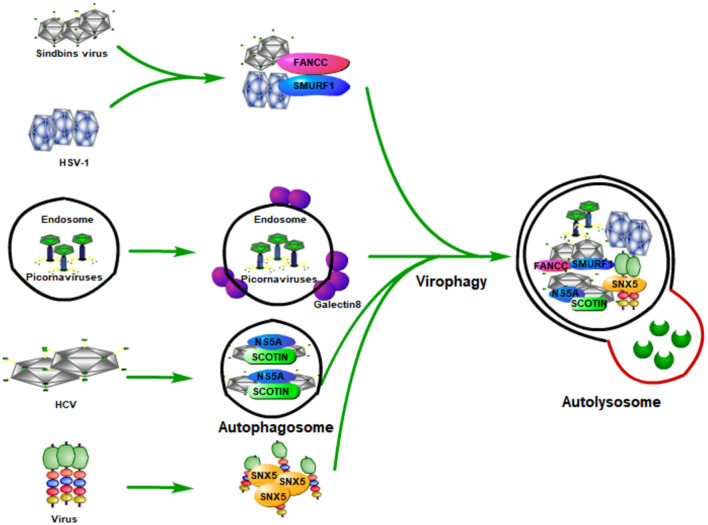
Host autophagy fights viral infection by selectively targeting viral particles to lysosome for degradation. During the viral infection, host autophagy can be inducted (known as virophagy), which can degrade viral particles, viral components, as well as host factors that are required by viruses for their replication. Some viruses are degraded by selective autophagy by being recognized by specific proteins of the host. Smad-Ubiquitin Regulatory Factor 1 (SMURF1), a HECT-domain ubiquitin ligase, and Fanconi anemia group C protein (FANCC) can target Sindbis capsid protein as well as the herpes simplex virus type 1 (HSV-1) to autophagosomes for virophagy, contributing to prevent viral infection. The invasion of the picornaviruses will puncture the endosomal membrane to release their genome into the host cytoplasm, causing the exposure of β-galactosides. Galectin-8 could specifically recognize β-galactosides and therefore mark the permeated endosomes for autophagic degradation. The endoplasmic reticulum (ER) protein SCOTIN can inhibit HCV replication by interacting with the HCV non-structural 5A (NS5A) protein, a critical factor for HCV replication, which can help to form autophagosomes for degradation and suppress infectious virion production in cells. Moreover, the endosomal protein sorting nexin 5 (SNX5) can target some viruses for virophagy, but not for basal level autophagy or stress-induced autophagy.

As a multi-step and tightly regulated cellular process for maintaining eukaryotic cellular homeostasis, autophagy is the only pathway that is able to degrade whole cellular organelles (such as mitochondria, peroxisomes, endoplasmic reticulum, nucleus, and liposomes) and various invading pathogens (including viruses) in either a selective or a non-selective manner (Khaminets et al., 2015; Mochida et al., 2015; Ammanathan et al., 2020; Jo et al., 2020; Miceli et al., 2020; Onishi et al., 2021). Upon viral infection, the induction of autophagy by viruses (known as virophagy) could be either proviral or antiviral (Dong and Levine, 2013; Delorme-Axford and Klionsky, 2019; Mijaljica and Klionsky, 2020). Virophagy plays its antiviral function probably through (1) selectively targeting viral particles to the lysosome for degradation (Figure 2), (2) promoting interferon production by activating host innate immune response, or (3) coordinating adaptive immunity by promoting antigen presentation.

### Host Autophagy Fights Viral Infection by Targeting Viral Particles to the Lysosome for Degradation

By delivering the invading pathogens to the lysosomes for degradation so as to discard them, autophagy is believed to be an important part of the host defense system. The process of degrading foreign microbial invaders by autophagy is known as xenophagy (Levine, 2005). The function of xenophagy to eliminate invading viruses, bacteria, fungi, or parasites, makes it an important immune player in pathogen infection. During viral infection, host autophagy can degrade viral particles, viral components, and the host factors required by viruses for their replication; therefore, host autophagy functions as a key innate antiviral response (Figure 2).

The first evidence of autophagy functions as antiviral means came from Sindbis viral infection. During its infection, overexpression of Beclin 1 (the mammalian Atg6 ortholog) in neurons could inhibit the Sindbis virus spread, reduce cellular apoptosis, and protect against fatal Sindbis virus encephalitis (Liang et al., 1998), whereas Atg5 (an essential component for the formation of autophagosomes in mammalian cells) deficiency in Sindbis-infected neurons leads to delayed clearance of viral proteins, accumulation of the p62 (also known as SQSTM1) adaptor protein, and increased cell death in neurons (Mizushima et al., 2001). Moreover, an *in vitro* study found that p62 could interact with Sindbis capsid protein and targets the viral capsid to the autophagosome (Orvedahl et al., 2010). Orvedahl et al. (2011) further found that Smad-Ubiquitin Regulatory Factor 1 (SMURF1), a HECT-domain ubiquitin ligase, is not required for general autophagy, but is needed by selective autophagy, such as virophagy and mitophagy. Moreover, they found that SMURF1 could interact with p62 and target Sindbis capsid to autophagosomes for virophagy. Fanconi anemia group C protein (FANCC) was also found to play an essential role in virophagy and mitophagy; its deficiency could inhibit the autophagic clearance of viruses (virophagy) and make mice more susceptible to lethal viruses (Sumpter et al., 2016). FANCC plays its role by interacting with the Sindbis capsid protein and therefore contributes to preventing viral infection (Minton, 2016; Sumpter et al., 2016). Besides Sindbis, SMURF1 and FANCC also inhibit HSV-1 infection through autophagy, indicating that these two proteins probably play virophagic factor functions during viral infection (Orvedahl et al., 2011; Sumpter et al., 2016).

Picornaviruses are archetypical non-enveloped viruses, which are a major cause of human and veterinary infections that lead to various diseases, such as polio and the common cold (Zhang et al., 2020). Host galectin 8 could detect picornaviruses and inhibit their infection through autophagy to degrade the viral genomic RNA (Staring et al., 2017). Specifically, the picornaviruses that enter the host will puncture the endosomal membrane and release their genome into the host cytoplasm, which causes the exposure of β-galactosides. Galectin-8 could specifically recognize β-galactosides and therefore marks the permeated endosomes for autophagic degradation.

As a small, enveloped RNA virus that mainly targets human hepatocytes, hepatitis C virus (HCV) is a major cause of liver cirrhosis and hepatocellular carcinoma worldwide (Zeisel et al., 2013; Kaplan, 2020). The prevention measures for HCV are absent and the current antiviral treatment for it is limited because of resistance, toxicity, and high costs (Zeisel et al., 2011; Hickman et al., 2015). Because of the discovery of HCV, the 2020 Nobel Prize in Physiology or Medicine has been awarded to Harvey J. Alter, Michael Houghton, and Charles M. Rice (Burki, 2020; Hoofnagle and Feinstone, 2020). Kim et al. (2016) found that the overexpression of endoplasmic reticulum (ER) protein SCOTIN inhibits HCV replication and infectious virion production in cells transfected with HCV. A further study found that SCOTIN could interact with HCV non-structural 5A (NS5A) protein, a critical factor for HCV replication, and target NS5A to autophagosomes for degradation. Moreover, inhibition of autophagy by silencing ATG7 or administering lysosomal inhibitors could relieve the suppressive effect of SCOTIN on HCV replication. They also showed that SCOTIN is merely a substrate for degradation of autophagy, but not affecting the whole process of autophagy; of note, the binding of transmembrane/proline-rich domain (TMPRD) of SCOTIN with Domain-II of NS5A is critically required for the trafficking of autophagosomal and NS5A degradation (Kim et al., 2016). These results suggest that autophagy restricts HCV replication through SCOTIN to target HCV NS5A protein to autophagosomes for degradation.

As a sensor of cytosolic DNA that activates the type I interferon pathway, cyclic GMP-AMP synthase (cGAS) could bind to microbial or self-DNA in the cytoplasm and therefore supervises infections or tissue damage (Sun et al., 2013). Specifically, cGAS is activated through binding with cytosolic DNA, which could further catalyze GTP and ATP to be cyclic GMP-AMP (cGAMP) (Wu et al., 2013). As a second messenger of cell, cGAMP could bind to and activate the stimulator of interferon genes (STING) (Ishikawa and Barber, 2008; Saitoh et al., 2009; Burdette et al., 2011; Wu et al., 2013; Zhang et al., 2013), which then recruits and activates the tank-binding kinase 1 (TBK1) causing the phosphorylation of the transcription factor IRF3 to induce the production of type I interferons and other cytokines (Cai et al., 2014; Liu et al., 2015; Zhang et al., 2019). Besides the important function in activating the immune response, STING could also activate autophagy during viral infections (Gui et al., 2019; Liu et al., 2019). Gui et al. (2019) showed that the binding of cGAMP with STING leads to the interaction of STING with SEC24C, causing the budding of STING from the endoplasmic reticulum into the COP-II vesicles and forming the endoplasmic reticulum–Golgi intermediate compartment (ERGIC), which functions as the membrane source for WIPI2 recruitment and LC3 lipidation, and finally causing the formation of autophagosomes targeting cytosolic DNA or DNA viruses to the lysosome for degradation (Gui et al., 2019). STING induces autophagy that is dependent on WIPI2 and ATG5, whereas other regulators of autophagy such as Beclin 1, Atg9a, ULK1, and p62 are not required (Gui et al., 2019; Liu et al., 2019). Interestingly, Gui et al. (2019) also showed that STING from the sea anemone is also able to induce autophagy but not interferons in response to the stimulation of cGAMP, which suggests that the cGAS-STING pathway-induced autophagy is probably an ancient and highly conserved mechanism that predates the emergence of the type I interferon pathway in vertebrates to eliminate the invading viruses.

How is autophagy induced in mammalian cells during viral infection? By using genome-wide short interfering RNA screens, the endosomal protein sorting nexin 5 (SNX5) is found to be required merely for virus-induced autophagy, but not for basal level autophagy or stress-induced autophagy (Dong et al., 2021). Deletion of SNX5 makes cultured cells more susceptible to viral infection, and mice deficient in SNX5 have a high lethality after infection with several human viruses (Dong et al., 2021). Moreover, they found that SNX5 could interact with PI3KC3-C1 and promote its activation at endosomes and therefore contributes to the initiation of autophagy during viral infection.

Collectively, host cells detect the invading viruses and activate the autophagy, which targets viruses to the lysosomes for degradation through the interaction of host protein with the viral protein. Future studies should focus on exploring additional viruses that can be selectively degraded by autophagy and clarifying the specific mechanisms involved in this process.

### Host Autophagy Defense Against Viral Infection by Promoting Interferon Production Through Activating the Innate Immune Response

**FIGURE 3:**
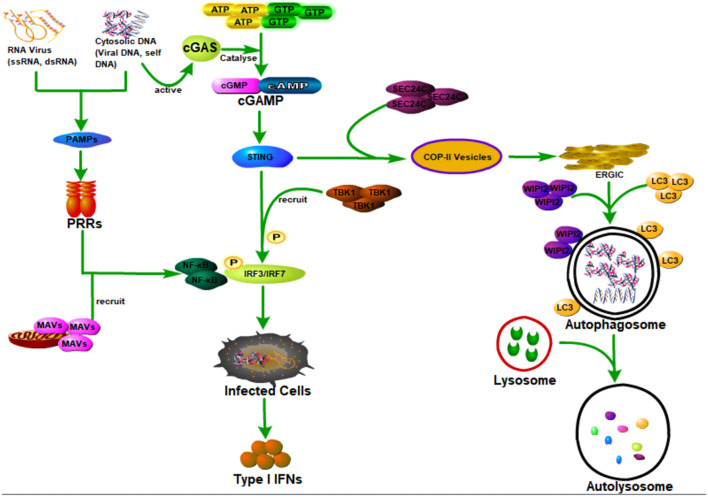
Host autophagy defense against viral infection by promoting interferon production through activating innate immune response. Cyclic GMP-AMP synthase (cGAS) can be activated when it is bound to the cytosolic DNA (microbial or self-DNA), then the activated cGAS can catalyze GTP and ATP to be cyclic GMP-AMP (cGAMP), which can bind to and activate the stimulator of interferon genes (STING). The binding of cGAMP with STING, on the one hand, can lead to the interaction of STING with SEC24C, causing the budding of STING from the ER into the COP-II vesicles and forming the endoplasmic reticulum–Golgi intermediate compartment (ERGIC), which functions as the membrane source for WIPI2 recruitment and LC3 lipidation, and finally causing the formation of autophagosomes targeting cytosolic DNA or DNA viruses to the lysosome for degradation. On the other hand, it can also recruit and activate the tank-binding kinase 1 (TBK1) causing the phosphorylation of the transcription factor IRF3 to induce the production of type I interferons (IFNs). In addition, viral DNA, viral double-stranded RNA (dsRNA), viral single-stranded RNA (ssRNA), or viral surface glycoproteins, express the pathogen-associated molecular patterns (PAMPs), which can be specifically recognized by several classes of germline-encoded pattern-recognition receptors (PRRs) of the host cells. Moreover, PRR could activate IRF3/IRF7 and NF-κB transcription factors by recruiting mitochondrial antiviral signaling protein (MAVS), leading to the activation of type I IFN responses and establishment of an antiviral state.

As the first line of defense against virus infection, the host innate immune system plays essential roles in recognizing invading viruses and inducing anti-viral responses to prevent viral invasion and pathogenesis before the generation of more specific protection by the adaptive immune system (Takeuchi and Akira, 2007; Koyama et al., 2008; Figure 3). The host innate immune system recognizes invading viruses by several classes of germline-encoded pattern-recognition receptors (PRRs), which could specifically recognize the pathogen-associated molecular patterns (PAMPs), such as viral DNA, viral double-stranded RNA (dsRNA), viral single-stranded RNA (ssRNA), or viral surface glycoproteins (Akira et al., 2006; Chan and Gack, 2016). The recognition of viral components by PRRs will induce the infected cells and other immune cells to produce type I interferons (IFNs) to aid in eliminating the invading viruses (Medzhitov, 2007).

There are several classes of PRRs with specific functions; virus-derived nucleic acids with distinct features are recognized by these specific host transmembrane or cytosol PRRs (Medzhitov, 2007; Gürtler and Bowie, 2013; Wu and Chen, 2014). As an important group of cytoplasmic RNA sensors, the RIG-I-like receptors (RLRs) are composed of three proteins (RIG-I, MDA5, and LGP2) that are similar in their structure and function, and all of them are able to recognize viral nucleic acid signatures during the viral infections (Rehwinkel and e Sousa, 2010; Bruns and Horvath, 2014). Recently, Hou et al. (2021) have found a novel selective autophagy receptor, CCDC50, which could deliver K63 polyubiquitination-activated RIG-I/MDA5 for degradation by lysosomes during viral infection and therefore negatively regulate the IFNs signaling pathway initiated by RLRs. As the best characterized class of PRR, toll-like receptors (TLRs) are transmembrane receptors that recognize viral nucleic acids within endosomal compartments (Medzhitov, 2007). Both of these two classes of PRR could activate IRF3/IRF7 and NF-κB transcription factors by recruiting mitochondrial antiviral signaling protein (MAVS), leading to the activation of type I interferon (IFN) responses and establishment of antiviral state (Seth et al., 2005; Akira et al., 2006; Medzhitov, 2007).

Lee et al. (2007) first elucidated the key function of autophagy in promoting interferon secretion in plasmacytoid dendritic cells (pDCs). They found that autophagy was required for the transportation of cytosolic viral into the lysosomes and the recognition of ssRNA viruses such as vesicular stomatitis virus (VSV) or Sendai virus (SeV) by TLR7. Moreover, they also found that autophagy was critically needed by pDCs during the production of interferon-α.

As an essential DNA virus sensor, cGAS could prompt IFNs production by generating cGAMP, which binds to and activates an endoplasmic reticulum-associated adaptor protein STING (Sun et al., 2013; Wu et al., 2013). Chen et al. (2016) have found that TRIM14 could block cGAS degradation through selective autophagy and therefore promote innate immune responses during viral infections. Specifically, upon the viral infections, TRIM14 could recruit USP14 to cut the lysine 48 (K48)-linked ubiquitin chains of cGAS at K414, causing inhibition of p62-mediated autophagic degradation of cGAS, therefore promoting the activation of type I interferon signaling to aid the elimination of the invading viruses (Chen et al., 2016).

These studies suggest that host autophagy plays an essential role in activating the innate immune response to eliminate the invading viruses by promoting the infected cells and other immune cells to produce IFNs. However, there are also some studies reporting that autophagy or its components contributed to the negative regulation of host innate immune response (Jounai et al., 2007; Lei et al., 2012; Liang et al., 2014; Jin et al., 2017; Du et al., 2018; Prabakaran et al., 2018). Therefore, during viral infection, the interaction between PRRs and autophagy resulted in the activation and/or inhibition of various host innate immune responses, causing first-rank antiviral effects.

### Host Autophagy Fights Viral Infection and Pathogenesis Through Coordinating Adaptive Immunity by Promoting Antigen Presentation

The efficient adaptive immune response is essential for the elimination of invading viruses. The first step of the adaptive immune response is the presentation of peptides of foreign or self-proteins on major histocompatibility complex (MHC) molecules at the cell surface of antigen-presenting cells (APCs) such as dendritic cells and macrophages, which then can be recognized by CD8^+^ or CD4^+^ T lymphocytes (Rammensee et al., 1993; Villadangos, 2001). In general, MHC class I (MHC-I) molecules specifically present antigenic peptides derived from intracellular proteins that have been digested by the proteasomal degradation system, whereas MHC class II (MHC-II) molecules specifically present antigenic peptides stemming from exogenous and membrane proteins that have been degraded by the endosomal/lysosomal system (Neefjes, 1999; Princiotta et al., 2003; Dengjel et al., 2005; Choi et al., 2018). However, there are certain situations where MHC-I molecules could present peptides stemming from exogenous antigens, which is a process called cross-presentation, mainly executed by a specific subset of dendritic cells (DCs) through endocytic and phagocytic pathways (Morón et al., 2004; Joffre et al., 2012; Blander, 2018; Muntjewerff et al., 2020). In addition, the peptides derived from intracellular proteins could also be loaded on MHC-II molecules through the process of autophagy (Nimmerjahn et al., 2003; Dörfel et al., 2005; Paludan et al., 2005; Münz, 2006).

#### Autophagy Contributes to the Intracellular Antigen Processing for MHC Class I Presentation

Macrophages infected with the Herpes simplex type 1 virus could activate the process of autophagy, which plays an essential role in the targeting of viral proteins to lysosomes and then loaded on MHC class I molecules for presentation (English et al., 2009b). During this process, a novel type of autophagosomes is involved, which is formed by coiling of the viral proteins enriched with nuclear membrane (English et al., 2009a). With the help of this process, the peptide derived from HSV-1 glycoprotein B (gB) could be presented to CD8^+^ T cells aided by proteasome function and the secretory pathway.

An endogenous human cytomegalovirus (HCMV) latency-associated protein, pUL138, could be presented by MHC-I through both the conventional TAP-dependent and the non-conventional TAP-independent pathways (Tey and Khanna, 2012). The TAP-dependent process uses the proteasomal machinery and ER-resident proteases of the conventional MHC class I pathway, whereas the TAP-independent process uses the vacuolar pathway mediated by autophagy. Importantly, this autophagy-mediated pathway is not dependent on proteasomal processing and Golgi transport, but dependent on the alternate cross-presentation pathway that only occurs within the endovacuolar compartment. Although this autophagy-mediated pathway uses minimal components of the conventional MHC-I machinery, it could generate and present the same peptide epitope as the conventional pathway.

#### Autophagy Promotes Intracellular Antigen Processing for MHC Class II Presentation

The MHC class II-positive cells such as dendritic, B, and epithelial cells constitutively form autophagosome, which then continuously fuse with multivesicular MHC class II-loading compartments to deliver cytoplasmic proteins for the presentation of MHC class II and antiviral immunity (Schmid et al., 2007). Endogenous Epstein–Barr virus nuclear antigen 1 (EBNA1) could be presented by MHC-II molecules and then recognized by CD4^+^ T cells (Paludan et al., 2005). Inhibition of lysosomal acidification led to the slow accumulation of EBNA1 in cytosolic autophagosomes. Moreover, blocking of autophagy using a PI3K inhibitor or by knockdown of ATG12 could decrease the presentation of EBNA1 to CD4^+^ T cells by MHC-II (Paludan et al., 2005).

#### Autophagy Contributes to the Extracellular Antigen Processing for MHC Class II Presentation

Herpes simplex virus (HSV) infection resulted in the impairment of CD4^+^ T-cell priming in Atg5-deficient mice in DCs, which succumbed to HSV infection (Lee et al., 2010). Moreover, autophagy is essential for the presentation of various phagocytosed antigens with TLR agonists, whereas autophagy is not required for innate immune recognition, antigen maturation, or cytokine production, as most of these processes remained intact in DCs deficient in Atg5 (Lee et al., 2010). These results suggest that autophagy plays an essential part in the processing and presentation of extracellular viral antigens by MHC-II in DCs.

#### Autophagy Also Contributes to Cross-Presentation of Extracellular, Endocytosed Antigens for MHC Class I Presentation

By presentation of extracellular, endocytosed antigens on MHC-I molecules, cross-presentation plays an essential role in the priming of CD8^+^ T-cell responses (Joffre et al., 2012). *In vivo*, subset-specific DCs are the major cells responsible for cross-presentation by adapting their endocytic and phagocytic pathways, which is essential for the immune defense against viruses and other intracellular pathogens (Blander, 2018; Theisen et al., 2018; Muntjewerff et al., 2020). On the one hand, autophagy could enhance cross-presentation by modulation of endosomes and assist the packaging of antigens released by donor cells, such as virus-infected cells, tumor cells, or dying cells, to neighbor DCs (Mintern et al., 2015; Dasari et al., 2016; Cruz et al., 2017); on the other hand, autophagy also contributed to the cross-presentation of viral antigens to CD8^+^ T cells during vaccination, which was facilitated through a stress-dependent initiation of autophagy in DCs (Ravindran et al., 2014). In addition, exocytosis mediated by autophagy could transfer extracellular antigens in LC3-coated autophagosome from the donor cells to DCs (Smed-Sörensen et al., 2012).

Thus, autophagy is involved in both the classical and the non-classical antigen presentation process, which is essential for the optimal processing and presentation of viral antigens. Moreover, the autophagy-mediated antigen presentation process is an important supplement to the conventional antigen presentation process; it may circumvent the various viral immune evasion strategies targeting the MHC-I or MHC-II machinery, therefore contributing to the elimination of invading viruses.

## Viruses Hijack and Subvert Host Autophagy to Aid Their Own Infections and Pathogenesis

**FIGURE 4:**
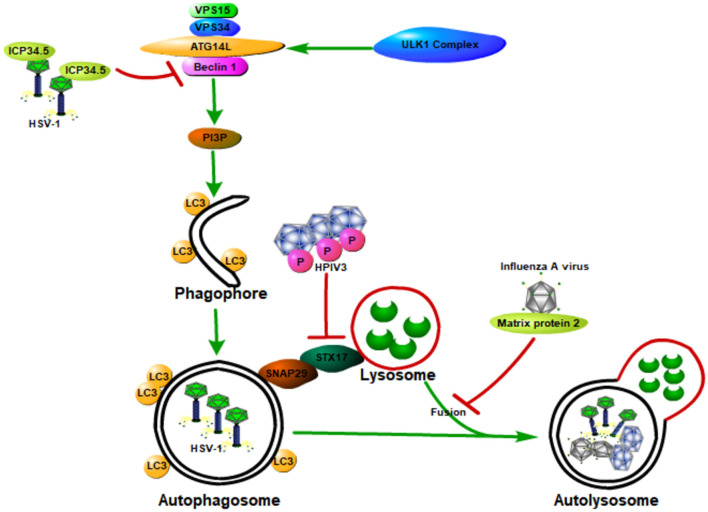
Viruses hijack and subvert host autophagy to aid their own infections and pathogenesis. Some viruses have developed various strategies to hijack and subvert host autophagy to aid their own infections and pathogenesis. These strategies include (1) directly inhibiting autophagy activation through blocking the function of host ATG proteins; (2) inhibiting autophagy downstream degradation pathway; and (3) subverting host autophagy to benefit viral replication. Class III PI3K complex I (PI3KC3-C1), which can produce PI3P, is necessary for the nucleation of autophagosomes and is composed of VPS34, VPS15, Beclin 1, and ATG14L. ICP34.5 expressed by the HSV-1 could inhibit host autophagy activation through binding with the host autophagy protein Beclin 1. The matrix protein 2 of influenza A virus could inhibit autophagy function by blocking the autophagosome–lysosome fusion, causing the enhanced virus-induced cell death of infected cells and elevated viral antigen release. The human parainfluenza virus type 3 (HPIV3) phosphoprotein (P) binds to SNAP29, blocking its binding to syntaxin 17, therefore inhibiting the fusion of autophagosome-lysosome mediated by these two host SNARE proteins. All of these will contribute to the viral infection and pathogenesis.

In general, host autophagy could inhibit viral replication, degrade viral particles, and activate host immune response, all of which contribute to the prevention of viral infection and pathogenesis. However, some viruses have developed various strategies to hijack and subvert host autophagy to aid their own infections and pathogenesis (Orvedahl and Levine, 2009a,b; Figure 4). These strategies include (1) directly inhibiting autophagy activation through blocking the function of host ATG proteins; (2) inhibiting autophagy downstream degradation pathway; and (3) subverting host autophagy to benefit for viral replication.

### Viruses Directly Inhibiting Autophagy Activation Through Blocking the Function of Host ATG Proteins

Neurovirulence protein ICP34.5, which is expressed by the herpes simplex virus type 1 (HSV-1), could inhibit host autophagy activation through binding with the host autophagy protein Beclin 1 (Orvedahl et al., 2007). HSV-1 virus deficient in the Beclin 1-binding domain of ICP34.5 is unable to block autophagy in neurons and shows a decrease in the ability to cause lethal encephalitis in mice. Deletion of PKR, an autophagy-inducing signaling molecule, could restore the neurovirulence of this Beclin 1-binding mutant virus (Orvedahl et al., 2007). These results suggest that the binding of Beclin 1 with ICP34.5 could inhibit host autophagy and contribute to viral neurovirulence, and the PKR is a Beclin 1 upstream effector during host defense against HSV-1. Moreover, the Us11 protein of HSV-1 could also inhibit host autophagy function by directly interacting with the PKR protein kinase, therefore contributing to the HSV-1 infection and pathogenesis (Lussignol et al., 2013).

### Viruses Counter Host Autophagy by Inhibiting the Autophagy Downstream Degradation Pathway

Matrix protein 2 of influenza A virus could inhibit autophagy function through blocking the autophagosome–lysosome fusion, causing the enhanced virus-induced cell death of infected cells and elevated viral antigen release (Gannagé et al., 2009; Rossman and Lamb, 2009). Human parainfluenza virus type 3 (HPIV3) could also lead to the incomplete autophagy of host cells through blocking autophagosome fusion with the lysosome, causing an increase in virus production (Ding et al., 2014). Specifically, the viral phosphoprotein (P) binds to SNAP29, blocking its binding to syntaxin 17 (STX17), therefore inhibiting autophagosome–lysosome fusion mediated by these two host SNARE proteins. Autophagosome accumulation due to incomplete autophagy could increase extracellular viral production but does not affect viral protein synthesis, therefore contributing to the viral infection and pathogenesis.

Severe Acute Respiratory Syndrome Coronavirus-2 (SARS-CoV-2), an enveloped, single-stranded and positive-sense RNA β-coronavirus, is the cause of the COVID-19 pandemic (Wang et al., 2020; Wu et al., 2020; Zhou et al., 2020). ORF3a, an accessory protein of SARS-CoV-2, could strongly inhibit autophagy activity by blocking the fusion of autophagosomes with lysosomes (Hayn et al., 2021; Miao et al., 2021; Qu et al., 2021; Zhang et al., 2021). Specifically, ORF3a is localized in the late endosome and directly interacts with and sequestrates VPS39, an essential component of the homotypic fusion and protein sorting (HOPS) complex, thereby preventing the HOPS complex from interacting with the STX17 or RAB7, which prevented the fusion machinery packaging, leading to an abnormal autophagosome–lysosome fusion. Moreover, SARS-CoV-2-expressed ORF3a and ORF7a can directly induce lysosomes injury and impair their function, such as inhibiting their acidification (Hayn et al., 2021; Koepke et al., 2021; Miao et al., 2021; Shroff and Nazarko, 2021). By doing this, SARS-CoV-2 could escape host lysosome degradation. Besides, the SARS-CoV-2 spike could hijack host autophagy to promote host cell inflammation and apoptosis probably through the ROS-suppressed PI3K/AKT/mTOR signaling (Li et al., 2021).

### Viruses Subvert Host Autophagy to Benefit Their Replication

As a DMV formed during autophagy, autophagosome provides a perfect place for the RNA viral replication through concentration of essential intermediates for viral package and protection of viral RNAs away from the detection by innate immune supervision and degradation. The host autophagy has been required by several viruses such as Coxsackieviruses (CVB) 3, CVB 4, foot and mouth disease virus, HCV, and poliovirus for their own replication, as genetic or pharmacological inhibition of autophagy could decrease viral yields (Taylor and Kirkegaard, 2008; Wong et al., 2008; Yoon et al., 2008; Dreux et al., 2009; O’Donnell et al., 2011). Moreover, some viruses such as dengue virus (DENV), usurp autophagy to enhance their replication by regulating cellular lipid metabolism (Heaton and Randall, 2010). Specifically, DENV infection induces the activation of lipophagy to release free fatty acids, causing an increase of cellular β-oxidation and more ATP generation, which contributes to the replication of DENV.

In summary, host autophagy is a double-edged sword during viral infection and pathogenesis. On the one hand, hosts could utilize their own autophagy to prevent viral infection and pathogenesis; on the other hand, viruses have evolved various strategies by which they hijack and subvert host autophagy to aid their infection and pathogenesis.

## Conclusion

As an evolutionarily conserved cellular process, autophagy is essential for both hosts and invading viruses. On the one hand, it could prevent viral infections and pathogenesis mainly by degrading viruses, initiating innate immune response, and facilitating antigen presentation. On the other hand, a mass of viruses have evolved strategies to hijack host autophagy for their own benefits. Although the function of host autophagy in viral infection and pathogenesis has been widely studied in the past two decades, current knowledge of autophagy in viral infections is still in its infancy and many important questions remain. For example, why some viruses have evolved strategies to evade host autophagy, whereas others have not? What is the driving force for this evolution? What roles do a variety of selective autophagy (such as mitophagy and lysophagy) play in viral infection and pathogenesis? What roles do microautophagy and chaperone-mediated autophagy play in viral infection and pathogenesis? Moreover, the specific function of autophagic proteins and the mechanisms controlling autophagy during viral infection and pathogenesis are still unclear. Further research is needed to elucidate the specific functions of different types of autophagy and the specific function in viral infections and pathogenesis, to develop more specific targeted drugs to combat epidemic viral infections, such as COVID-19.

## Author Contributions

J-XT and H-FL formulated and conceived the study. Y-SW, SL, D-YL, J-XT, and H-FL wrote the manuscript. All authors helped to interpret results and approved the final version of the manuscript.

## Conflict of Interest

The authors declare that the research was conducted in the absence of any commercial or financial relationships that could be construed as a potential conflict of interest.

## Publisher’s Note

All claims expressed in this article are solely those of the authors and do not necessarily represent those of their affiliated organizations, or those of the publisher, the editors and the reviewers. Any product that may be evaluated in this article, or claim that may be made by its manufacturer, is not guaranteed or endorsed by the publisher.

## References

[B1] Akira S. Uematsu S. Takeuchi O. (2006). Pathogen recognition and innate immunity. *Cell* 124 783–801. 10.1016/j.cell.2006.02.015 16497588

[B2] Ammanathan V. Mishra P. Chavalmane A. K. Muthusamy S. Jadhav V. Siddamadappa C. (2020). Restriction of intracellular *Salmonella* replication by restoring TFEB-mediated xenophagy. *Autophagy* 16 1584–1597. 10.1080/15548627.2019.1689770 31744366 PMC8386624

[B3] Blander J. M. (2018). Regulation of the cell biology of antigen cross-presentation. *Annu. Rev. Immunol.* 36 717–753. 10.1146/annurev-immunol-041015-055523 29490164 PMC6430635

[B4] Bruns A. M. Horvath C. M. (2014). Antiviral RNA recognition and assembly by RLR family innate immune sensors. *Cytokine Growth Factor Rev.* 25 507–512. 10.1016/j.cytogfr.2014.07.006 25081315 PMC4252791

[B5] Burdette D. L. Monroe K. M. Sotelo-Troha K. Iwig J. S. Eckert B. Hyodo M. (2011). STING is a direct innate immune sensor of cyclic di-GMP. *Nature* 478 515–518. 10.1038/nature10429 21947006 PMC3203314

[B6] Burki T. (2020). Nobel prize for hepatitis C virus discoverers. *Lancet* 396:1058. 10.1016/S0140-6736(20)32111-5 33038954

[B7] Cai X. Chiu Y.-H. Chen Z. J. (2014). The cGAS-cGAMP-STING pathway of cytosolic DNA sensing and signaling. *Mol. Cell* 54 289–296. 10.1016/j.molcel.2014.03.040 24766893

[B8] Chan Y. K. Gack M. U. (2016). Viral evasion of intracellular DNA and RNA sensing. *Nat. Rev. Microbiol.* 14 360–373. 10.1038/nrmicro.2016.45 27174148 PMC5072394

[B9] Chen M. Meng Q. Qin Y. Liang P. Tan P. He L. (2016). TRIM14 inhibits cGAS degradation mediated by selective autophagy receptor p62 to promote innate immune responses. *Mol. Cell* 64 105–119. 10.1016/j.molcel.2016.08.025 27666593

[B10] Choi Y. Bowman J. W. Jung J. U. (2018). Autophagy during viral infection—a double-edged sword. *Nat. Rev. Microbiol.* 16 341–354. 10.1038/s41579-018-0003-6 29556036 PMC6907743

[B11] Cruz F. M. Colbert J. D. Merino E. Kriegsman B. A. Rock K. L. (2017). The biology and underlying mechanisms of cross-presentation of exogenous antigens on MHC-I molecules. *Annu. Rev. Immunol.* 35 149–176. 10.1146/annurev-immunol-041015-055254 28125356 PMC5508990

[B12] Dasari V. Rehan S. Tey S. K. Smyth M. J. Smith C. Khanna R. (2016). Autophagy and proteasome interconnect to coordinate cross-presentation through MHC class I pathway in B cells. *Immunol. Cell Biol.* 94 964–974. 10.1038/icb.2016.59 27297581

[B13] Delorme-Axford E. Klionsky D. J. (2019). *Inflammatory-Dependent Sting Activation Induces Antiviral Autophagy to Limit Zika Virus in the Drosophila Brain.* Milton Park: Taylor & Francis. 10.1080/15548627.2018.1539585 PMC6287683 30354937

[B14] Dengjel J. Schoor O. Fischer R. Reich M. Kraus M. Müller M. (2005). Autophagy promotes MHC class II presentation of peptides from intracellular source proteins. *Proc. Natl. Acad. Sci. U.S.A.* 102 7922–7927. 10.1073/pnas.0501190102 15894616 PMC1142372

[B15] Deretic V. Saitoh T. Akira S. (2013). Autophagy in infection, inflammation and immunity. *Nat. Rev. Immunol.* 13 722–737. 10.1038/nri3532 24064518 PMC5340150

[B16] Ding B. Zhang G. Yang X. Zhang S. Chen L. Yan Q. (2014). Phosphoprotein of human parainfluenza virus type 3 blocks autophagosome-lysosome fusion to increase virus production. *Cell Host Microbe* 15 564–577. 10.1016/j.chom.2014.04.004 24832451

[B17] Dong X. Levine B. (2013). Autophagy and viruses: adversaries or allies? *J. Innate Immun.* 5 480–493. 10.1159/000346388 23391695 PMC3790331

[B18] Dong X. Yang Y. Zou Z. Zhao Y. Ci B. Zhong L. (2021). Sorting nexin 5 mediates virus-induced autophagy and immunity. *Nature* 589 456–461. 10.1038/s41586-020-03056-z 33328639 PMC7856200

[B19] Dörfel D. Appel S. Grünebach F. Weck M. M. Müller M. R. Heine A. (2005). Processing and presentation of HLA class I and II epitopes by dendritic cells after transfection with in vitro–transcribed MUC1 RNA. *Blood* 105 3199–3205. 10.1182/blood-2004-09-3556 15618468

[B20] Dreux M. Gastaminza P. Wieland S. F. Chisari F. V. (2009). The autophagy machinery is required to initiate hepatitis C virus replication. *Proc. Natl. Acad. Sci. U.S.A.* 106 14046–14051. 10.1073/pnas.0907344106 19666601 PMC2729017

[B21] Du Y. Duan T. Feng Y. Liu Q. Lin M. Cui J. (2018). LRRC25 inhibits type I IFN signaling by targeting ISG15-associated RIG-I for autophagic degradation. *EMBO J.* 37 351–366. 10.15252/embj.201796781 29288164 PMC5793803

[B22] English L. Chemali M. Duron J. Rondeau C. Laplante A. Gingras D. (2009b). Autophagy enhances the presentation of endogenous viral antigens on MHC class I molecules during HSV-1 infection. *Nat. Immunol.* 10 480–487. 10.1038/ni.1720 19305394 PMC3885169

[B23] English L. Chemali M. Desjardins M. (2009a). Nuclear membrane-derived autophagy, a novel process that participates in the presentation of endogenous viral antigens during HSV-1 infection. *Autophagy* 5 1026–1029. 10.4161/auto.5.7.9163 19556870

[B24] Feng Y. He D. Yao Z. Klionsky D. J. (2014). The machinery of macroautophagy. *Cell Res.* 24 24–41. 10.1038/cr.2013.168 24366339 PMC3879710

[B25] Gannagé M. Dormann D. Albrecht R. Dengjel J. Torossi T. Rämer P. C. (2009). Matrix protein 2 of influenza A virus blocks autophagosome fusion with lysosomes. *Cell Host Microbe* 6 367–380. 10.1016/j.chom.2009.09.005 19837376 PMC2774833

[B26] Gui X. Yang H. Li T. Tan X. Shi P. Li M. (2019). Autophagy induction via STING trafficking is a primordial function of the cGAS pathway. *Nature* 567 262–266. 10.1038/s41586-019-1006-9 30842662 PMC9417302

[B27] Gürtler C. Bowie A. G. (2013). Innate immune detection of microbial nucleic acids. *Trends Microbiol.* 21 413–420. 10.1016/j.tim.2013.04.004 23726320 PMC3735846

[B28] Hamasaki M. Furuta N. Matsuda A. Nezu A. Yamamoto A. Fujita N. (2013). Autophagosomes form at ER–mitochondria contact sites. *Nature* 495 389–393. 10.1038/nature11910 23455425

[B29] Hayn M. Hirschenberger M. Koepke L. Nchioua R. Straub J. H. Klute S. (2021). Systematic functional analysis of SARS-CoV-2 proteins uncovers viral innate immune antagonists and remaining vulnerabilities. *Cell Rep.* 35:109126. 10.1016/j.celrep.2021.109126 33974846 PMC8078906

[B30] Heaton N. S. Randall G. (2010). Dengue virus-induced autophagy regulates lipid metabolism. *Cell Host Microbe* 8 422–432. 10.1016/j.chom.2010.10.006 21075353 PMC3026642

[B31] Hickman M. De Angelis D. Vickerman P. Hutchinson S. Martin N. (2015). Hcv treatment as prevention in people who inject drugs–testing the evidence. *Curr. Opin. Infect Dis.* 28:576. 10.1097/QCO.0000000000000216 26524330 PMC4659818

[B32] Hoofnagle J. H. Feinstone S. M. (2020). The discovery of Hepatitis C—the 2020 Nobel prize in physiology or medicine. *N. Engl. J. Med.* 383 2297–2299. 10.1056/NEJMp2031110 33283991

[B33] Hou P. Yang K. Jia P. Liu L. Lin Y. Li Z. (2021). A novel selective autophagy receptor, CCDC50, delivers K63 polyubiquitination-activated RIG-I/MDA5 for degradation during viral infection. *Cell Res.* 31 62–79. 10.1038/s41422-020-0362-1 32612200 PMC7852694

[B34] Ishikawa H. Barber G. N. (2008). STING is an endoplasmic reticulum adaptor that facilitates innate immune signalling. *Nature* 455 674–678. 10.1038/nature07317 18724357 PMC2804933

[B35] Jin S. Tian S. Luo M. Xie W. Liu T. Duan T. (2017). Tetherin suppresses type I interferon signaling by targeting MAVS for NDP52-mediated selective autophagic degradation in human cells. *Mol. Cell* 68 308–322. e304. 10.1016/j.molcel.2017.09.005 28965816

[B36] Jo D. S. Park S. J. Kim A.-K. Park N. Y. Kim J. B. Bae J.-E. (2020). Loss of HSPA9 induces peroxisomal degradation by increasing pexophagy. *Autophagy* 16 1989–2003. 10.1080/15548627.2020.1712812 31964216 PMC7595578

[B37] Joffre O. P. Segura E. Savina A. Amigorena S. (2012). Cross-presentation by dendritic cells. *Nat. Rev. Immunol.* 12 557–569. 10.1038/nri3254 22790179

[B38] Jounai N. Takeshita F. Kobiyama K. Sawano A. Miyawaki A. Xin K.-Q. (2007). The Atg5–Atg12 conjugate associates with innate antiviral immune responses. *Proc. Natl. Acad. Sci. U.S.A.* 104 14050–14055. 10.1073/pnas.0704014104 17709747 PMC1955809

[B39] Kaplan D. E. (2020). Hepatitis C virus. *Ann. Intern. Med.* 173 ITC33–ITC48. 10.7326/AITC202009010 32866406

[B40] Kaushik S. Cuervo A. M. (2012). Chaperone-mediated autophagy: a unique way to enter the lysosome world. *Trends Cell Biol.* 22 407–417. 10.1016/j.tcb.2012.05.006 22748206 PMC3408550

[B41] Khaminets A. Heinrich T. Mari M. Grumati P. Huebner A. K. Akutsu M. (2015). Regulation of endoplasmic reticulum turnover by selective autophagy. *Nature* 522 354–358. 10.1038/nature14498 26040720

[B42] Kim N. Kim M.-J. Sung P. S. Bae Y. C. Shin E.-C. Yoo J.-Y. (2016). Interferon-inducible protein SCOTIN interferes with HCV replication through the autolysosomal degradation of NS5A. *Nat. Commun.* 7:10631. 10.1038/ncomms10631 26868272 PMC4754343

[B43] Koepke L. Hirschenberger M. Hayn M. Kirchhoff F. Sparrer K. M. J. A. (2021). Manipulation of autophagy by SARS-CoV-2 proteins. *Autophagy* 1–3. 10.1080/15548627.2021.1953847 [Epub ahead of print]. 34281462 PMC8496524

[B44] Koyama S. Ishii K. J. Coban C. Akira S. (2008). Innate immune response to viral infection. *Cytokine* 43 336–341. 10.1016/j.cyto.2008.07.009 18694646

[B45] Kuma A. Hatano M. Matsui M. Yamamoto A. Nakaya H. Yoshimori T. (2004). The role of autophagy during the early neonatal starvation period. *Nature* 432 1032–1036. 10.1038/nature03029 15525940

[B46] Lamb C. A. Yoshimori T. Tooze S. A. (2013). The autophagosome: origins unknown, biogenesis complex. *Nat. Rev. Mol. Cell Biol.* 14 759–774. 10.1038/nrm3696 24201109

[B47] Lee H. K. Lund J. M. Ramanathan B. Mizushima N. Iwasaki A. (2007). Autophagy-dependent viral recognition by plasmacytoid dendritic cells. *Science* 315 1398–1401. 10.1126/science.1136880 17272685

[B48] Lee H. K. Mattei L. M. Steinberg B. E. Alberts P. Lee Y. H. Chervonsky A. (2010). In vivo requirement for Atg5 in antigen presentation by dendritic cells. *Immunity* 32 227–239. 10.1016/j.immuni.2009.12.006 20171125 PMC2996467

[B49] Lei Y. Wen H. Yu Y. Taxman D. J. Zhang L. Widman D. G. (2012). The mitochondrial proteins NLRX1 and TUFM form a complex that regulates type I interferon and autophagy. *Immunity* 36 933–946. 10.1016/j.immuni.2012.03.025 22749352 PMC3397828

[B50] Levine B. (2005). Eating oneself and uninvited guests: autophagy-related pathways in cellular defense. *Cell* 120 159–162. 10.1016/S0092-8674(05)00043-7 15680321

[B51] Levine B. Kroemer G. (2008). Autophagy in the pathogenesis of disease. *Cell* 132 27–42. 10.1016/j.cell.2007.12.018 18191218 PMC2696814

[B52] Levine B. Kroemer G. (2019). Biological functions of autophagy genes: a disease perspective. *Cell* 176 11–42. 10.1016/j.cell.2018.09.048 30633901 PMC6347410

[B53] Levine B. Mizushima N. Virgin H. W. (2011). Autophagy in immunity and inflammation. *Nature* 469 323–335. 10.1038/nature09782 21248839 PMC3131688

[B54] Li F. Li J. Wang P.-H. Yang N. Huang J. Ou J. (2021). SARS-CoV-2 spike promotes inflammation and apoptosis through autophagy by ROS-suppressed PI3K/AKT/mTOR signaling. *Biochim. Biophys. Acta Mol. Basis Dis.* 1867:166260. 10.1016/j.bbadis.2021.166260 34461258 PMC8390448

[B55] Liang Q. Seo G. J. Choi Y. J. Kwak M.-J. Ge J. Rodgers M. A. (2014). Crosstalk between the cGAS DNA sensor and Beclin-1 autophagy protein shapes innate antimicrobial immune responses. *Cell Host Microbe* 15 228–238. 10.1016/j.chom.2014.01.009 24528868 PMC3950946

[B56] Liang X. H. Kleeman L. K. Jiang H. H. Gordon G. Goldman J. E. Berry G. (1998). Protection against fatal Sindbis virus encephalitis by beclin, a novel Bcl-2-interacting protein. *J. Virol.* 72 8586–8596. 10.1128/JVI.72.11.8586-8596.1998 9765397 PMC110269

[B57] Liu D. Wu H. Wang C. Li Y. Tian H. Siraj S. (2019). STING directly activates autophagy to tune the innate immune response. *Cell Death Differ.* 26 1735–1749. 10.1038/s41418-018-0251-z 30568238 PMC6748081

[B58] Liu S. Cai X. Wu J. Cong Q. Chen X. Li T. (2015). Phosphorylation of innate immune adaptor proteins MAVS, STING, and TRIF induces IRF3 activation. *Science* 347:aaa2630. 10.1126/science.aaa2630 25636800

[B59] Lussignol M. Queval C. Bernet-Camard M.-F. Cotte-Laffitte J. Beau I. Codogno P. (2013). The herpes simplex virus 1 Us11 protein inhibits autophagy through its interaction with the protein kinase PKR. *J. Virol.* 87 859–871. 10.1128/JVI.01158-12 23115300 PMC3554085

[B60] Medzhitov R. (2007). Recognition of microorganisms and activation of the immune response. *Nature* 449 819–826. 10.1038/nature06246 17943118

[B61] Melia T. J. Lystad A. H. Simonsen A. (2020). Autophagosome biogenesis: from membrane growth to closure. *J. Cell Biol.* 219:e202002085. 10.1083/jcb.202002085 32357219 PMC7265318

[B62] Miao G. Zhao H. Li Y. Ji M. Chen Y. Shi Y. (2021). ORF3a of the COVID-19 virus SARS-CoV-2 blocks HOPS complex-mediated assembly of the SNARE complex required for autolysosome formation. *Dev. Cell* 56 427–442. e425. 10.1016/j.devcel.2020.12.010 33422265 PMC7832235

[B63] Miceli C. Roccio F. Penalva-Mousset L. Burtin M. Leroy C. Nemazanyy I. (2020). The primary cilium and lipophagy translate mechanical forces to direct metabolic adaptation of kidney epithelial cells. *Nat. Cell Biol.* 22 1091–1102. 10.1038/s41556-020-0566-0 32868900

[B64] Mijaljica D. Klionsky D. J. (2020). *Autophagy/Virophagy: A “Disposal Strategy” to Combat COVID-19.* Milton Park: Taylor & Francis. 10.1080/15548627.2020.1782022 PMC7751505 32578486

[B65] Mijaljica D. Prescott M. Devenish R. J. (2011). Microautophagy in mammalian cells: revisiting a 40-year-old conundrum. *Autophagy* 7 673–682. 10.4161/auto.7.7.14733 21646866

[B66] Mintern J. D. Macri C. Chin W. J. Panozza S. E. Segura E. Patterson N. L. (2015). Differential use of autophagy by primary dendritic cells specialized in cross-presentation. *Autophagy* 11 906–917. 10.1080/15548627.2015.1045178 25950899 PMC4502655

[B67] Minton K. (2016). Autophagy: inflammatory pathology of Fanconi anaemia. *Nat. Rev. Mol. Cell Biol.* 17 330–331. 10.1038/nrm.2016.64 27165789

[B68] Mizushima N. Levine B. (2010). Autophagy in mammalian development and differentiation. *Nat. Cell Biol.* 12 823–830. 10.1038/ncb0910-823 20811354 PMC3127249

[B69] Mizushima N. Levine B. (2020). Autophagy in human diseases. *N. Engl. J. Med.* 383 1564–1576. 10.1056/NEJMra2022774 33053285

[B70] Mizushima N. Levine B. Cuervo A. M. Klionsky D. J. (2008). Autophagy fights disease through cellular self-digestion. *Nature* 451 1069–1075. 10.1038/nature06639 18305538 PMC2670399

[B71] Mizushima N. Yamamoto A. Hatano M. Kobayashi Y. Kabeya Y. Suzuki K. (2001). Dissection of autophagosome formation using Apg5-deficient mouse embryonic stem cells. *J. Cell Biol.* 152 657–668. 10.1083/jcb.152.4.657 11266458 PMC2195787

[B72] Mochida K. Oikawa Y. Kimura Y. Kirisako H. Hirano H. Ohsumi Y. (2015). Receptor-mediated selective autophagy degrades the endoplasmic reticulum and the nucleus. *Nature* 522 359–362. 10.1038/nature14506 26040717

[B73] Morón G. Dadaglio G. Leclerc C. (2004). New tools for antigen delivery to the MHC class I pathway. *Trends Immunol.* 25 92–97. 10.1016/j.it.2003.11.008 15102368

[B74] Muntjewerff E. M. Meesters L. D. Van Den Bogaart G. (2020). Antigen cross-presentation by macrophages. *Front. Immunol.* 11:1276. 10.3389/fimmu.2020.01276 32733446 PMC7360722

[B75] Münz C. (2006). Autophagy and antigen presentation. *Cell. Microbiol.* 8 891–898. 10.1111/j.1462-5822.2006.00714.x 16681832

[B76] Nakatogawa H. (2020). Mechanisms governing autophagosome biogenesis. *Nat. Rev. Mol. Cell Biol.* 21 439–458. 10.1038/s41580-020-0241-0 32372019

[B77] Neefjes J. (1999). CIIV, MIIC and other compartments for MHC class II loading. *Eur. J. Immunol.* 29 1421–1425. 10.1002/(SICI)1521-4141(199905)29:05<1421::AID-IMMU1421>3.0.CO;2-C 10359095

[B78] Nimmerjahn F. Milosevic S. Behrends U. Jaffee E. M. Pardoll D. M. Bornkamm G. W. (2003). Major histocompatibility complex class II-restricted presentation of a cytosolic antigen by autophagy. *Eur. J. Immunol.* 33 1250–1259. 10.1002/eji.200323730 12731050

[B79] O’Donnell V. Pacheco J. M. LaRocco M. Burrage T. Jackson W. Rodriguez L. L. (2011). Foot-and-mouth disease virus utilizes an autophagic pathway during viral replication. *Virology* 410 142–150. 10.1016/j.virol.2010.10.042 21112602 PMC7126820

[B80] Onishi M. Yamano K. Sato M. Matsuda N. Okamoto K. (2021). Molecular mechanisms and physiological functions of mitophagy. *EMBO J.* 40:e104705. 10.15252/embj.2020104705 33438778 PMC7849173

[B81] Orenstein S. J. Cuervo A. M. (2010). Chaperone-mediated autophagy: molecular mechanisms and physiological relevance. *Semin. Cell Dev. Biol.* 21 719–26. 10.1016/j.semcdb.2010.02.005 20176123 PMC2914824

[B82] Orvedahl A. Alexander D. Tallóczy Z. Sun Q. Wei Y. Zhang W. (2007). HSV-1 ICP34. 5 confers neurovirulence by targeting the beclin 1 autophagy protein. *Cell Host Microbe* 1 23–35. 10.1016/j.chom.2006.12.001 18005679

[B83] Orvedahl A. Levine B. (2009a). Autophagy in mammalian antiviral immunity. *Curr. Top Microbiol. Immunol.* 335 267–285. 10.1007/978-3-642-00302-8_13 19802570

[B84] Orvedahl A. Levine B. (2009b). Eating the enemy within: autophagy in infectious diseases. *Cell Death Differ.* 16 57–69. 10.1038/cdd.2008.130 18772897 PMC2736877

[B85] Orvedahl A. MacPherson S. Sumpter R. Jr. Tallóczy Z. Zou Z. Levine B. (2010). Autophagy protects against Sindbis virus infection of the central nervous system. *Cell Host Microbe* 7 115–127. 10.1016/j.chom.2010.01.007 20159618 PMC2860265

[B86] Orvedahl A. Sumpter R. Xiao G. Ng A. Zou Z. Tang Y. (2011). Image-based genome-wide siRNA screen identifies selective autophagy factors. *Nature* 480 113–117. 10.1038/nature10546 22020285 PMC3229641

[B87] Paludan C. Schmid D. Landthaler M. Vockerodt M. Kube D. Tuschl T. (2005). Endogenous MHC class II processing of a viral nuclear antigen after autophagy. *Science* 307 593–596. 10.1126/science.1104904 15591165

[B88] Prabakaran T. Bodda C. Krapp C. Zhang B. C. Christensen M. H. Sun C. (2018). Attenuation of c GAS-STING signaling is mediated by a p62/SQSTM 1-dependent autophagy pathway activated by TBK1. *EMBO J.* 37:e97858. 10.15252/embj.201797858 29496741 PMC5897779

[B89] Princiotta M. F. Finzi D. Qian S.-B. Gibbs J. Schuchmann S. Buttgereit F. (2003). Quantitating protein synthesis, degradation, and endogenous antigen processing. *Immunity* 18 343–354. 10.1016/S1074-7613(03)00051-7 12648452

[B90] Qu Y. Wang X. Zhu Y. Wang W. Wang Y. Hu G. (2021). ORF3a-mediated incomplete autophagy facilitates severe acute respiratory syndrome coronavirus-2 replication. *Front. Cell Dev. Biol.* 9:716208. 10.3389/fcell.2021.716208 34386498 PMC8353283

[B91] Rammensee H.-G. Falk K. Rötzschke O. (1993). MHC molecules as peptide receptors. *Curr. Opin. Immunol.* 5 35–44. 10.1016/0952-7915(93)90078-7 7680871

[B92] Ravindran R. Khan N. Nakaya H. I. Li S. Loebbermann J. Maddur M. S. (2014). Vaccine activation of the nutrient sensor GCN2 in dendritic cells enhances antigen presentation. *Science* 343 313–317. 10.1126/science.1246829 24310610 PMC4048998

[B93] Rehwinkel J. e Sousa C. R. (2010). RIGorous detection: exposing virus through RNA sensing. *Science* 327 284–286. 10.1126/science.1185068 20075242

[B94] Rossman J. S. Lamb R. A. (2009). Autophagy, apoptosis, and the influenza virus M2 protein. *Cell Host Microbe* 6 299–300. 10.1016/j.chom.2009.09.009 19837369

[B95] Sahu R. Kaushik S. Clement C. C. Cannizzo E. S. Scharf B. Follenzi A. (2011). Microautophagy of cytosolic proteins by late endosomes. *Dev. Cell* 20 131–139. 10.1016/j.devcel.2010.12.003 21238931 PMC3025279

[B96] Saitoh T. Fujita N. Hayashi T. Takahara K. Satoh T. Lee H. (2009). Atg9a controls dsDNA-driven dynamic translocation of STING and the innate immune response. *Proc. Natl. Acad. Sci. U.S.A.* 106 20842–20846. 10.1073/pnas.0911267106 19926846 PMC2791563

[B97] Schmid D. Pypaert M. Münz C. (2007). Antigen-loading compartments for major histocompatibility complex class II molecules continuously receive input from autophagosomes. *Immunity* 26 79–92. 10.1016/j.immuni.2006.10.018 17182262 PMC1805710

[B98] Seth R. B. Sun L. Ea C.-K. Chen Z. J. (2005). Identification and characterization of MAVS, a mitochondrial antiviral signaling protein that activates NF-κB and IRF3. *Cell* 122 669–682. 10.1016/j.cell.2005.08.012 16125763

[B99] Shibutani S. T. Yoshimori T. (2014). A current perspective of autophagosome biogenesis. *Cell Res.* 24 58–68. 10.1038/cr.2013.159 24296784 PMC3879706

[B100] Shroff A. Nazarko T. Y. J. C. (2021). The molecular interplay between human coronaviruses and autophagy. *Cells* 10:2022. 10.3390/cells10082022 34440791 PMC8392315

[B101] Smed-Sörensen A. Chalouni C. Chatterjee B. Cohn L. Blattmann P. Nakamura N. (2012). Influenza A virus infection of human primary dendritic cells impairs their ability to cross-present antigen to CD8 T cells. *PLoS Pathog.* 8:e1002572. 10.1371/journal.ppat.1002572 22412374 PMC3297599

[B102] Staring J. von Castelmur E. Blomen V. A. van den Hengel L. G. Brockmann M. Baggen J. (2017). PLA2G16 represents a switch between entry and clearance of picornaviridae. *Nature* 541 412–416. 10.1038/nature21032 28077878

[B103] Sumpter R. Jr. Sirasanagandla S. Fernández ÁF. Wei Y. Dong X. Franco L. (2016). Fanconi anemia proteins function in mitophagy and immunity. *Cell* 165 867–881. 10.1016/j.cell.2016.04.006 27133164 PMC4881391

[B104] Sun L. Wu J. Du F. Chen X. Chen Z. J. (2013). Cyclic GMP-AMP synthase is a cytosolic DNA sensor that activates the type I interferon pathway. *Science* 339 786–791. 10.1126/science.1232458 23258413 PMC3863629

[B105] Takeuchi O. Akira S. (2007). Recognition of viruses by innate immunity. *Immunol. Rev.* 220 214–224. 10.1111/j.1600-065X.2007.00562.x 17979849

[B106] Taylor M. P. Kirkegaard K. (2008). Potential subversion of autophagosomal pathway by picornaviruses. *Autophagy* 4 286–289. 10.4161/auto.5377 18094610

[B107] Tey S.-K. Khanna R. (2012). Autophagy mediates transporter associated with antigen processing-independent presentation of viral epitopes through MHC class I pathway. *Blood* 120 994–1004. 10.1182/blood-2012-01-402404 22723550

[B108] Theisen D. J. Davidson J. T. Briseño C. G. Gargaro M. Lauron E. J. Wang Q. (2018). WDFY4 is required for cross-presentation in response to viral and tumor antigens. *Science* 362 694–699. 10.1126/science.aat5030 30409884 PMC6655551

[B109] Villadangos J. A. (2001). Presentation of antigens by MHC class II molecules: getting the most out of them. *Mol. Immunol.* 38 329–346. 10.1016/S0161-5890(01)00069-4 11684289

[B110] Wang C. Horby P. W. Hayden F. G. Gao G. F. J. T. (2020). A novel coronavirus outbreak of global health concern. *Lancet* 395 470–473. 10.1016/S0140-6736(20)30185-9 31986257 PMC7135038

[B111] Wong J. Zhang J. Si X. Gao G. Mao I. McManus B. M. (2008). Autophagosome supports coxsackievirus B3 replication in host cells. *J. Virol.* 82 9143–9153. 10.1128/JVI.00641-08 18596087 PMC2546883

[B112] Wu F. Zhao S. Yu B. Chen Y.-M. Wang W. Song Z.-G. (2020). A new coronavirus associated with human respiratory disease in China. *Nature* 579 265–269. 10.1038/s41586-020-2008-3 32015508 PMC7094943

[B113] Wu J. Chen Z. J. (2014). Innate immune sensing and signaling of cytosolic nucleic acids. *Annu. Rev. Immunol.* 32 461–488. 10.1146/annurev-immunol-032713-120156 24655297

[B114] Wu J. Sun L. Chen X. Du F. Shi H. Chen C. (2013). Cyclic GMP-AMP is an endogenous second messenger in innate immune signaling by cytosolic DNA. *Science* 339 826–830. 10.1126/science.1229963 23258412 PMC3855410

[B115] Yoon S. Y. Ha Y. E. Choi J. E. Ahn J. Lee H. Kweon H.-S. (2008). Coxsackievirus B4 uses autophagy for replication after calpain activation in rat primary neurons. *J. Virol.* 82 11976–11978. 10.1128/JVI.01028-08 18799585 PMC2583648

[B116] Zeisel M. B. Felmlee D. J. Baumert T. F. (2013). Hepatitis C virus entry. Hepatitis C virus: from molecular virology to antiviral therapy. *Curr. Top Microbiol. Immunol.* 369 87–112. 10.1007/978-3-642-27340-7_4 23463198

[B117] Zeisel M. B. Fofana I. Fafi-Kremer S. Baumert T. F. (2011). Hepatitis C virus entry into hepatocytes: molecular mechanisms and targets for antiviral therapies. *J. Hepatol.* 54 566–576. 10.1016/j.jhep.2010.10.014 21146244

[B118] Zhang C. Shang G. Gui X. Zhang X. Bai X.-C. Chen Z. J. (2019). Structural basis of STING binding with and phosphorylation by TBK1. *Nature* 567 394–398. 10.1038/s41586-019-1000-2 30842653 PMC6862768

[B119] Zhang X. Paget M. Wang C. Zhu Z. Zheng H. (2020). Innate immune evasion by picornaviruses. *Eur. J. Immunol.* 50 1268–1282. 10.1002/eji.202048785 32767562

[B120] Zhang X. Shi H. Wu J. Zhang X. Sun L. Chen C. (2013). Cyclic GMP-AMP containing mixed phosphodiester linkages is an endogenous high-affinity ligand for STING. *Mol. Cell* 51 226–235. 10.1016/j.molcel.2013.05.022 23747010 PMC3808999

[B121] Zhang Y. Sun H. Pei R. Mao B. Zhao Z. Li H. (2021). The SARS-CoV-2 protein ORF3a inhibits fusion of autophagosomes with lysosomes. *Cell Discov.* 7:31. 10.1038/s41421-021-00268-z 33947832 PMC8096138

[B122] Zhou P. Yang X.-L. Wang X.-G. Hu B. Zhang L. Zhang W. (2020). A pneumonia outbreak associated with a new coronavirus of probable bat origin. *Nature* 579 270–273. 10.1038/s41586-020-2012-7 32015507 PMC7095418

